# Supplementary material for: Amphibian mast cells serve as barriers to chytrid fungus infections
Source: eLife. 2024 Jul 31;12:RP92168. doi: 10.7554/eLife.92168 (PMC11290838; doi:10.7554/eLife.92168)
Supplement: Supplementary file 1. [file elife-92168-supp1.docx]

**Supplementary file 1. List of primer sequences**

| Target Name | Primer Sequence (5’🡪 3’) |
| --- | --- |
| *Bd* *its1* sense | GCCATATGTCACGAGTCGAA |
| *Bd* *its1* antisense | GCCAAGAGATCCGTTGTCA |
| *gapdh* sense | GACACTCACTCCTCCATCTTTG |
| *gapdh* antisense | TGCTGTAGCCGCATTCATTA |
| *hdc* sense | GAATCTGAAAGCTGGGAGAGAA |
| *hdc* antisense | GATGTCAGGGCAGGAAAGTAG |
| *il4* sense | GACATCAAGGACACCTGAAGAA |
| *il4* antisense | GTCACAGGGAATCGGTACTAAAC |
| *mag* sense | GGCCTTTGCAGATGAAGATTTAG |
| *mag* antisense | CTACTGCTTCTGCATCTCGTT |
| *pgla* sense | CTGCACTCTGTGCAACCATAA |
| *pgla* antisense | CTCCAGCTTTAGATGCCATTCC |
